# A general framework for analyzing tumor subclonality using SNP array and DNA sequencing data

**DOI:** 10.1186/s13059-014-0473-4

**Published:** 2014-09-25

**Authors:** Bo Li, Jun Z Li

**Affiliations:** Program of Bioinformatics, University of Michigan, 5940A Buhl, Box 5618, Ann Arbor, MI 48109-5618 USA; Department of Human Genetics, University of Michigan, 5940A Buhl, Box 5618, Ann Arbor, MI 48109-5618 USA

## Abstract

Intra-tumor heterogeneity reflects cancer genome evolution and provides key information for diagnosis and treatment. When bulk tumor tissues are profiled for somatic copy number alterations (sCNA) and point mutations, it may be difficult to estimate their cellular fractions when a mutation falls within a sCNA. We present the Clonal Heterogeneity Analysis Tool, which estimates cellular fractions for both sCNAs and mutations, and uses their distributions to inform macroscopic clonal architecture. In a set of approximately 700 breast tumors, more than half appear to contain multiple recognizable aneuploid tumor clones, and many show subtype-specific differences in clonality for known cancer genes.

**Electronic supplementary material:**

The online version of this article (doi:10.1186/s13059-014-0473-4) contains supplementary material, which is available to authorized users.

## Background

It has been recognized for nearly 40 years that cancer is a dynamic disease and its evolution follows a classical Darwinian process [1,2]. After the proposal of the two-hit model of oncogenesis [3], and especially after the discovery of the linear progression from benign polyps to colorectal cancer via a series of mutational events [4,5], it was briefly envisioned that cancer could be understood in most cases by simply finding the small number of events that act sequentially to drive step-wise clonal selection. However, initial efforts to sequence most coding genes in tumor DNA revealed remarkable heterogeneity between tumors in each cancer type examined [6-9]: typically, very few (<10) genes are mutated in >10% of tumors, but many (40 to 80) are mutated in 1% to 5% of tumors. Further, heterogeneity in cancer could manifest on other levels: not just among different patients, but also among tumors of different grades or organ sites in the same patient, as well as among different cells within a tumor [10,11]. Heterogeneity at any of these levels could confound diagnosis and treatment, and underlie the inherent evasiveness of this disease. Most genomic analyses to date, notably those led by the Cancer Genome Atlas (TCGA) Research Network [12-15] and the International Cancer Genome Consortium (ICGC) [16] have focused on inter-tumor heterogeneity. These studies analyze hundreds of tumors per cancer type, relying on bulk tissue samples, usually for one sample per patient. The data were primarily interpreted by regarding each tumor as a single population of cells with uniform character. Despite the inherent limitation of this assumption, as shown by the widely reported tumor-normal mixing [17-19], large-scale inter-tumor comparisons have led to important new insights into significantly mutated genes [12,13], recurrently perturbed pathways [20], mutation signatures [16,21], tumor subtypes [22,23], molecular predictors of outcome, and commonalities or distinctions among different cancer types [24]. However, these studies are not designed to adequately investigate intra-tumor heterogeneity. Ultimately, cancer genome evolution takes place at the single-cell level, and it is the cellular complexity and its dynamics that give rise to both intra- and inter-tumor heterogeneity. Currently, cytogenetic methods are of low throughput and often cannot assure representative sampling. And the cost of single-cell sequencing [25-28] remains prohibitively expensive for all but the proof-of-concept studies. Under such constraints, many groups have surveyed intra-tumor heterogeneity by comparing multiple specimens from the same patient by longitudinal sampling or spatial sampling (mainly for solid tumors). Almost invariably, analyses of longitudinal samples have uncovered dramatic temporal changes of the cancer cell population that often correlate with disease progression, severity, and treatment resistance [29-32]. Similarly, multi-region comparisons have revealed extensive genomic variability across different geographic sectors of the tumor [33,34], or between the primary and metastatic tumors [35]. These studies, while using samples collected with a higher spatial or temporal resolution than those in TCGA and ICGC, often still contain heterogeneous populations of cells [35-37].

Fortunately, while bulk tissue data describe the global average of multiple subpopulations of cells, it is sometimes possible to statistically infer the number and genomic profile of such subpopulations. For example, when a sample is sequenced deeply, the somatic mutation frequencies sometimes cluster around a small number of distinct frequency ‘modes’ [38,39], suggesting that somatic mutations of similar frequencies may reside in the same population of cells and these cells may have descended from the same founder cell. For this reason, these mutations are said to belong to the same ‘clone’ or ‘subclones’, the latter referring to a clonal population of a relatively small cellular fraction. This inference task, essentially a deconvolution problem (or Blind Source Separation Problem), presents many analytical challenges, since both the number of subclones and the genomic profile of each need to be estimated simultaneously, and somatic copy number alterations (sCNAs) and somatic single-nucleotide variants (SNVs) often reside in the same region yet have unknown phase or genealogical order. Currently available methods often need to invoke simplifying assumptions and often focus on a subset of the issues. For example, *ABSOLUTE* [40] uses sCNA data to estimate the global mixing ratio of aneuploid and euploid cells, but only under a tumor-normal, two-population assumption, which involves a single tumor population of full clonality. When a sCNA or SNV is subclonal, *ABSOLUTE* makes the qualitative designation of ‘subclonal’ without quantitatively estimating the clonality. Other methods also invoke other types of compromises, and we will defer the description of these limitations to the Discussion.

**Figure 1:**
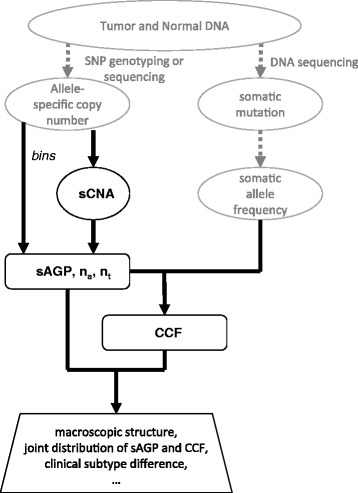
**Schematics of** ***CHAT*** **pipeline.** Tumor and Normal DNA samples are profiled for allele-specific copy number alterations by SNP arrays and somatic mutations by DNA sequencing. Gray texts and broken arrows (in the upper portion of the figure) indicate input data. *CHAT* offers two options to partitions the genome: by naturally identified sCNAs or by predefined bins. It then estimates sAGP for each partition (left side). Inference of CCF and timing-phase scenarios relies on sAGP of sCNA, copy number configuration (n_b_, n_t_), and SAF of the mutation (right side). CCF and sAGP can be used in a wide range of downstream analyses (bottom).

In this work, we developed Clonal Heterogeneity Analysis Tool (*CHAT*) as a general framework for estimating the cellular frequencies of both sCNAs and SNVs. It is suitable for analyzing genomewide SNP genotyping and DNA sequencing data for tumor-normal pairs (Figure 1). *CHAT* begins by identifying regions of sCNA or by partitioning the genome into bins; and for each sCNA or bin, it estimates a local mixing ratio, called segmental aneuploid genome proportion (sAGP), between a euploid population and a single aneuploid population carrying the local CNA. The assumption of local two-way mixing does not imply there are only two cell populations globally. It is akin to the infinite-site model in population genetics, stating that each locus experienced only one copy number alteration, without a second over-riding alteration or the reversal to the original germline state (that is, back mutation). After calculating sAGP for every sCNA in the tumor, *CHAT* estimates the cellular prevalence of SNVs (also called cancer cell fraction, or CCF, as in [32]) by adjusting the observed somatic allele frequency (SAF) from sequencing data according to the background copy number status, while also considering the sCNA clonality (sAGP), the relative order of occurrence between the SNV and its associated sCNA, and their cis- or trans-relationship. Through simulation we show that *CHAT* performs well in quantitatively recovering sAGP, CCF, and the underlying evolutionary scenario. We also show that it estimates CCF more accurately than *EXPANDS* and *PyClone* in most scenarios and CNA states. We have applied *CHAT* to calculate sAGP for sCNAs, and CCF for SNVs, across 732 human breast tumor samples previously analyzed for inter-tumor diversity by TCGA [14] (Materials and methods, Data access and sCNA identification), and we will present two vignettes of the results. Lastly, we discuss the model identifiability issue and compare the theoretical features of *CHAT* with that of several similar methods.

## Results

### Estimation of sAGP for sCNAs

The simplest form of intra-tumor heterogeneity is normal cell ‘contamination’, that is, mixture of aneuploid cells in the tumor with euploid cells in the surrounding normal tissue, the latter carrying the full and balanced set of chromosomes found in germline DNA. In our previous work [18], we developed a method to calculate the overall fraction of the tumor cells, termed Aneuploid Genomic Proportion (AGP), assuming the global mixing of a tumor and a normal population. In brief, allelic intensity data from SNP genotyping arrays (or DNA sequencing) provide copy number information of the two parental chromosomes: n_a_ and n_b_. Since n_a_ and n_b_ are both integers, the logarithm of total intensity ratio, LRR ~ log(n_a_ + n_b_), and the observed B allele frequency, BAF = n_b_/(n_a_ + n_b_), adopt a finite number of discrete BAF- LRR combinations for different CNAs, and reside in ‘canonical positions’ in the BAF-LRR plot. When aneuploid cells are mixed with euploid cells, logR-BAF positions of tumor sCNAs ‘contract’ towards the euploid position; and different mixing ratios result in different degrees of contraction. Based on this feature we can quantitatively estimate a genome-wide tumor mixing ratio [18]. Our algorithm relies on the same type of information, and shares the same goal, as several other methods (for example, *ASCAT* and *ABSOLUTE*) [17,40]. All of these methods assume that there is a single tumor population and use the combined information from all CNAs.

**Figure 2:**
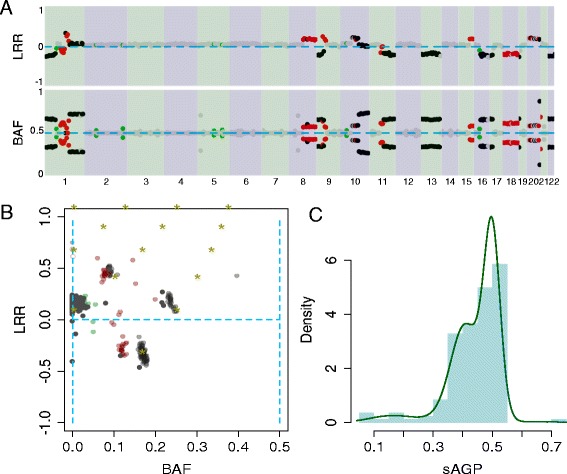
**Example of intra-tumor heterogeneity (breast tumor sample TCGA-A1-A0SD). (A)** BAF and LRR tracks for binned segments, showing different levels of contraction along the genome. Segments shown in the same color (black, red, green) have similar cellular fractions, and each may represent events in the same clonal population. **(B)** BAF-LRR plot for the same sample, showing different levels of contraction for segments of different colors. **(C)** MCMC fitting of sAGP distribution supports three modes, with peaks around sAGP =0.5, 0.4, and 0.2. The distribution of sAGP is indicated by the light blue histogram, while the fitted three-Gaussian density is shown in dark green.

However, intra-tumor heterogeneity may also manifest as the co-existence of multiple tumor cell populations, each with its own copy number profile [41]. One example is shown in Figure 2A, where the sCNA segments marked in red show stronger contractions to the diploid track, for both LRR and BAF, than those marked in black; whereas those marked in green show even stronger contractions (Figure 2A and B). As mentioned above, since all the sCNAs in black have similar cellular fraction values, we may infer the existence of a subclone, defined as a subpopulation of cells carrying the same set of events (the ‘black’ sCNAs) due to their descent from a common ancestor tumor cell. This is the most parsimonious explanation why different somatic events in the genome could reach the same frequency. Meanwhile, another set of events, such as those in red, show a different cellular fraction values, suggesting the existence of a second subclone. Note that a subclone may be nested in a parental clone, and carry the events that are ‘older’ and of higher frequency. These ‘parental events’ may be shared between two sibling clones, each carrying its own unique set of newer events. Thus the sibling clones are disjoint (that is, non-overlapping) population of cells even when they share some events by common descent. Since the sCNA segments with different mixing ratios are interspersed along the genome, this regional variation of clonality motivates us to extend the earlier concept of genome-wide AGP to a new, segment-specific measure: sAGP.

A previous method [41] has attempted to simultaneously estimate the number of subpopulations, the copy number profile for each, and their mixing ratios. This deconvolution problem can be solved, in principle, via a general convex optimization algorithm, but in practice it is limited by computation burden, which increases quickly for more than several dozen events or more than three to four populations. Our method takes an alternative approach: *CHAT* estimates the mixing ratio for each sCNA (or bin) separately, postponing the question as to which events might belong to the same subclone by virtue of clustering around a similar sAGP, and how many subclones there might be. Thus, *CHAT* decouples the inference of local sAGPs from the subsequent clustering of sAGP, and is vastly more efficient: its computation time scales linearly with the number of sCNA events and there is nearly no time penalty when needing to consider an increasing number of subpopulations.

The estimation of sAGP follows a similar approach as estimating the global AGP [18], relying on the degree of contraction of each sCNA (Figure 2B, Materials and methods, sAGP inference). The method has the implicit assumption that at each sCNA the mixing involves only two populations, one of which is euploid. This assumption is largely satisfied when the somatic genome has experienced relatively sparse copy number changes, without global doubling or multiple rounds of complex local aberration. In effect, it assumes that, even though different sCNAs in the genome may belong to multiple populations of aneuploid cells, at each sCNA region there is only one aneuploid state that is mixed with the euploid state. As such, sAGP is a local quantity inferred for each sCNA, and is naturally assigned zero in regions with no sCNA. The input can be either SNP array data or sequencing data as long as there is a sufficient density of sites with allele-specific copy number data. At this step there is no need to determine if a sCNA is clonal or subclonal. Also of note is that in *ABSOLUTE* [40], subclones CNA events were described by a global purity value and a real-valued copy fraction (not an integer copy number). In contrast, *CHAT* explicitly models the mixing of a euploid population and an aneuploid population, involving a real-valued local mixing ratio and integer copy numbers.

### Macroscopic clonal structure

Before describing the next step in *CHAT* - using sAGP and the observed SAF values to estimate CCF - we introduce an important downstream inference based on the genome-wide distribution of sAGP values. When there are a sufficient number of sCNAs or bins covered by sCNA, *CHAT* produces a sufficient number of sAGP values; and their distribution could inform the clonal structure of the tumor. First, for some tumors the sAGP histogram may contain a single peak, potentially accompanied by a flat (nearly uniform) background distribution. This pattern can arise in a tumor containing a single clone that cover a large fraction of the sCNA-bearing portion of the genome, potentially with many other clones that cover much smaller portions of the genome and they are undiscernible in the sAGP spectrum. Second, for other tumors the histogram may follow a multi-modal distribution, representing a number of distinct clusters of somatic events, each with a different sAGP, with each cluster covering a comparable portion of the genome as to be recognizable in the histogram (an example is shown in Figure 2C).

In all, there are three attributes of each sAGP histogram. (1) The number of the identifiable modes corresponds to the number of identifiable cell populations. (2) The position of each mode denotes the cellular frequency of the sCNAs in each cluster, and reflects the clonality of the cell populations carrying these sCNAs. The right-most peak represents the sCNAs with the highest sAGP values; and they suggest the existence of a population of cells with the highest cellular fraction in the tumor. This population is typically called the dominant clone. The peaks to the left represent sCNAs with lower sAGP values and they are carried by populations of cells with lower cellular fractions. These populations are often called subclone 1, subclone 2, and so on, but they may be nested within the dominant clone, and also carry the sCNAs in the right-most peak. (3) The areas under the peaks reflect the number of the sCNAs, or the regularly spaced bins, that belong to each peak. Note that the right-most peak may not have the largest area, thus the dominant clone may not carry sCNAs that cover the widest portion of the genome.

There are at least two ways to define the spatial unit in the sAGP analysis, and *CHAT* provides both options (Materials and methods, Data access and sCNA identification). The first is to calculate sAGP for regularly spaced bins, either for a fixed window width or for a fixed number of SNPs. The resulting sAGP values resemble the conventional genetic ‘markers’; and each tumor has a guaranteed number and density of such markers to construct the sAGP histogram, which is interpreted analogously to the allele frequency spectrum in population genetics studies. However, the bins do not match the naturally occurring sCNAs, which are highly variable in lengths, from tens of kb to entire chromosome arms. The sCNAs shorter than the bin width would have their true sAGP values ‘diluted’ by flanking euploid segments in the same bin; whereas those longer than the bin width would generated a string of correlated sAGP values as the same sCNA is artificially divided into multiple adjacent bins, thus violating the assumption that sAGPs are independent. In the second option, *CHAT* will apply the identified sCNA as the naturally occurring spatial unit for sAGP calculation. While this has the advantage that all sAGPs are truly independent, there are two disadvantages. First, the longer (or shorter) sCNAs provide more (or less) precise estimates of sAGP, but this information of confidence was discarded, as it is also the case in [41]. Two, there will be large tumor-tumor variations in the number of sCNAs, and some tumors may not have enough sCNAs to construct an informative histogram for estimating clonal composition. In short, the per-bin sAGPs (option 1) are derived from segments of similar length and have similar confidence intervals - they are identically distributed but not independent random variables. Conversely, the per-sCNA sAGPs (option 2) are independent, but are not identically distributed due to varying lengths. Rigorously speaking, neither is suited for analyzing macroscopic clonal architecture but can be applied in exploratory analysis, especially when there is no other data type such as the SNVs (see below).

When the primary goal of using *CHAT* is to accurately estimate CCF, which relies on accurate sAGP values, the user is advised to calculate sAGP using sCNAs as the spatial unit rather than the bins. Alternatively, when the primary goal is to explore clonal composition of a tumor, and if there are too few sCNAs and if most of them are very large, it is beneficial to increase the number of informative features, just as the detection of population stratification requires many ancestry informative markers. Here the user may choose regularly spaced bins to increase the number of available sAGPs. In fact, when sCNAs are few and large, it is more advisable to collect sequencing data; and if the mutation rate is high and/or the entire genome is sequenced (as opposed to small targeted regions), the number of SNVs may exceed that of sCNAs, and it is better to rely on the CCF histogram to estimate clonal structure. CCF distributions have the important advantage of meeting the condition of independent and identically distributed variable. Ultimately, the best approach is to integrate the sAGP and CCF distributions in estimating clonal structure.

*CHAT* fits the uni-modal pattern with a maximal likelihood framework, and the multi-modal pattern using a Bayesian Monte-Carlo Markov Chain (MCMC) approach, with Dirichlet Process prior to estimate a hierarchical Gaussian mixture model [42]. The approach is similar to those introduced in [32,38,39,43]. Details are provided in Materials and methods, Statistical modeling to infer macroscopic clonal structure. Model selection is based on the Bayesian Information Criterion (BIC) [44]. In Discussion we will further interpret the uni-modal and multi-modal patterns in terms of the likely evolutionary dynamics and the relationship to classic concepts such as punctuated equilibrium [45] and episodic evolution.

### Estimating cell fractions of somatic mutations

#### Nature of the problem

The next step of *CHAT* turns from estimating sAGP of sCNAs to estimating the frequency of cells carrying a specific mutation, that is, single nucleotide variant (SNV) or small insertion/deletion (indel). Here the method addresses the case where the tumor DNA has been sequenced, either for the whole genome or for a targeted subset, such as the exome. The input of the analysis is the observed number of reads in the sequence data containing the mutation as well as those containing the un-mutated allele. The relative fraction of mutation-bearing reads is termed somatic allele frequency (SAF). Following [32], we adopt CCF to denote the percentage of cells in the tumor sample carrying a specific somatic mutation. CCF is also termed cellular prevalence in [43]. The task is to use the observed SAF to estimate the unknown CCF.

If the mutation resides in a normal diploid region, it typically occurs on the background of one of the two parental chromosomes, contributing to about half the sequence reads in this region. In this simple case, as the fraction of cells carrying the mutation is CCF, the expected fraction of sequence reads carrying the mutation, SAF, is simply a binomial variable with an expected value of CCF/2. We therefore can estimate CCF by SAF × 2. However, if the mutation resides in a sCNA, the relationship between CCF and SAF depends on the copy number configuration (for example, copy neutral loss of heterozygosity (CN-LOH), deletion, amplification, and so on) and its sAGP. Further, it also depends on the chromosomal background in which the mutation occurs. For example, in a region of heterozygous amplification where one of the chromosomes has been duplicated, if the mutation occurs on the duplicated chromosome, it will contribute twice the number of sequence reads than the case where it occurs on the un-duplicated chromosome. Lastly, if the mutation occurs after the duplication has happened and the duplication-bearing clone is undergoing expansion, only a subset of the duplication-bearing cells will carry the mutation, and the relative size of this subpopulation can be any value in 0 to 100% and will also affect the relationship between CCF and SAF. In the following we systematically consider these possible scenarios. We will make the parsimonious assumption that each mutation only occurred once in the evolutionary history of the tumor cell population, therefore we will ignore the possibility of recurrent mutation at the same position, or simultaneous emergence of the same mutation is different subpopulations of cells. We will treat SNVs and indels equally, and use the term ‘mutation’ to denote both.

#### Order-phase scenarios between sCNA and SNV

For a somatic mutation revealed by tumor DNA sequencing, with an observed SAF value, we consider the task of estimating CCF if this mutation resides in an sCNA, and the sCNA has been discovered by either SNP array genotyping data [17,40] or by sequencing data [32,38]. We assume that the sCNA has been well characterized, such that we already know n_a_ and n_b_, the copy number of its major and minor alleles, respectively, that is, n_a_ ≥ n_b_, and n_t_ = n_a_ + n_b_ is the total copy number. We also assume that its sAGP has been calculated using the method described above, and that SAF has been corrected for known sequencing errors and local biases [21,46]. Below we present the CCF estimation procedure for the case of heterozygous amplification (n_a_ =2, n_b_ =1). The two other common sCNA types, heterozygous deletion (n_a_ =1, n_b_ =0) and CN-LOH (n_a_ =2, n_b_ =0), are described in Materials and methods, CCF estimation and scenario identifiability for CN-LOH and deletion.

**Figure 3:**
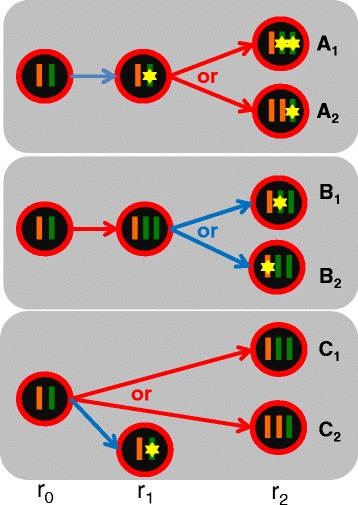
**Lineage scenarios for a mutation that fall in a region of heterozygous amplification.** In scenario **A**_**1**_, the mutation (yellow star) occurred before the amplification, which doubled the mutation-bearing chromosome (shown in green). The three illustrated populations have fractions of r_0_, r_1_ and r_2_, which sum up to 1. **A**_**2**_ is similar to **A**_**1**_, except that the un-mutated chromosome (in orange) was doubled. For scenario **B**, the amplification happened first, and the mutation occurred either on the amplified (**B**_**1**_) or the unamplified (**B**_**2**_) allele. For scenario **C**, the mutation and the sCNA occurred on independent lineages, and the amplification affects either the same (**C**_**1**_) or the opposite chromosome (**C**_**2**_) as the mutation. Blue arrow: mutation occurrence; red arrow: sCNA occurrence.

When a mutation resides in a sCNA region, there are three main scenarios that describe the possible mutation-sCNA combinations in terms of their relative temporal order and the chromosomal background of the mutation (Figure 3):

A. The mutation and sCNA emerged sequentially, with the mutation occurring first, and the sCNA occurring in a subset of mutation-bearing cells (Figure 3A). This led to the co-existence of three subpopulations: the original euploid mutation-free cells, with the population fraction of r_0_; cells carrying the mutation only, with a fraction of r_1_; and cells carrying both the mutation and the sCNA (r_2_). The last subpopulation has two alternative outcomes: **A**_**1**_: the duplication occurred on the mutation-bearing chromosome, and **A**_**2**_: the duplication occurred on the mutation-free chromosome. Intuitively, **A**_**1**_ will have higher SAF than **A**_**2**_ with the same (r_0_, r_1_, r_2_) fractions.
B. Like **A**, the mutation and sCNA emerged sequentially; but unlike A, the sCNA occurred first, with the mutation occurring in a subset of sCNA-bearing cells (Figure 3B). Again we have three subpopulations: the original cells (r_0_), cells carrying only the sCNA (r_1_) and those carrying both (r_2_). The last subpopulation has two alternatives: mutation occurring on one of the duplicated chromosome (**B**_**1**_) or the un-duplicated chromosome (**B**_**2**_).
C. The mutation and sCNA emerged independently, that is, appearing in non-overlapping populations of cells (Figure 3C). This also led to three subpopulations: the original cells (r_0_), cells carrying only the mutation (r_1_) and those carrying only the sCNA (r_2_). Note that we do not consider the fourth population that carries both the mutation and the sCNA. This outcome would require that the mutation occurred twice, once in the original cells and again in the sCNA-bearing cells. Or it requires the sCNA to occur twice. Under the Maximal Parsimonious assumption, recurrent appearance of the same mutation or the same sCNA is highly unlikely in the same tumor.

The three scenarios outlined above covered all the possible mutation-sCNA combinations for one-copy amplification without recurrence. In Additional file 1: Figure S1 and Materials and methods, CCF estimation and scenario identifiability for CN-LOH and deletion, we show that heterozygous deletion and CN-LOH involve similar scenarios **A**, **B** and **C**, and each leads to a similar set of three subpopulations as described by r_0_, r_1_, and r_2_, with r_0_ + r_1_ + r_2_ = 1.

#### CCF as a function of sAGP, SAF and the underlying scenario

When the (n_a_, n_b_) configuration and evolutionary scenario is known, CCF can be estimated from: (1) the pre-estimated sAGP of the sCNA (denoted *p* hereafter for simplicity) on which the mutation occurs; and (2) the observed allele frequency, SAF, of the somatic mutation (denoted *f* hereafter). In the following we derive the CCF estimation procedure for heterozygous duplication (n_a_ =2, n_b_ =1), and leave CN-LOH (n_a_ =2, n_b_ =0), and deletion (n_a_ =1, n_b_ =0) to Materials and methods, CCF estimation and scenario identifiability for CN-LOH and deletion.

For amplification, in scenario **A**_**1**_, n_t_ =3, the average total copy number *n*_*t*_ =2 × (1 - *p*) + *n*_*t*_ × *p* =2 + *p*. The sAGP *p* = *r*_2_. The SAF *f* = (*r*_1_ + 2*r*_2_)/(2 + *p*). This led to the expression *r*_1_ = *f* × (2 + *p*) - 2*r*_2_. Since *CCF* = *r*_1_ + *r*_2_, we have

1 $$ CC{F}^{A_1}\left(f,{n}_b,{n}_t,p\right)=f\times \left(2+p\right)-{r}_2=f\times \left(2+p\right)-p $$

In **A**_**2**_, the situation is similar to **A**_**1**_ except that *f* = (*r*_1_ + *r*_2_)/(2 + *p*). This led to *r*_1_ = *f* × (2 + *p*) - *r*_2_, and

2 $$ CC{F}^{A_2}\left(f,{n}_b,{n}_t,p\right)=f\times \left(2+p\right) $$

In **B**_**1**_**and B**_**2**_, the sAGP: *p* = *r*_1_ + *r*_2_. The SAF: *f* = *r*_2_/(2 + *p*). This led to *r*_2_ = *f* × (2 + *p*). Since *CCF* = *r*_2_, we have

3 $$ CC{F}^B\left(f,{n}_b,{n}_t,p\right)=f\times \left(2+p\right) $$

In **C**_**1**_**and C**_**2**_, the sAGP: *p* = *r*_2_. The SAF: *f* = *r*_1_/(2 + *p*). This led to *r*_1_ = *f* × (2 + *p*). Since *CCF* = *r*_1_, we have

4 $$ CC{F}^C\left(f,{n}_b,{n}_t,p\right)=f\times \left(2+p\right) $$

Note that equations (2), (3), and (4) are identical. Thus even if we do not know how to distinguish among scenarios **A**_**2**_, **B**, and **C**, CCF still has the same dependency on sAGP and SAF, and can be estimated as long as we can recognize **A**_**1**_ and **A**_**2**_/**B/C**. Thus CCF identifiability is easier to achieve than scenario identifiability.

Similar expressions for CN-LOH and deletion are presented in Materials and methods, CCF estimation and scenario identifiability for CN-LOH and deletion. In the general copy number configuration of n_a_ and n_b_, for scenarios A_1_, A_2_, B, and C we have

5 $$ CC{F}^{A_1}\left(f,{n}_b,{n}_t,p\right)={n}_t\times f-p\times {n}_a+p $$

6 $$ CC{F}^{A_2}\left(f,{n}_b,{n}_t,p\right)={n}_t\times f-p\times {n}_b+p $$

7 $$ CC{F}^{B/C}\left(f,{n}_b,{n}_t,p\right)={n}_t\times f $$

with *n*_*t*_ =2 × (1 - *p*) + *n*_*t*_ × *p*, is the averaged copy number at the locus.

Thus, for a given pair of mutation and sCNA, with known SAF and sAGP values, once we know which scenario applies we can use Equations (1), (2), (3), (4), (5), (6), and (7) to estimate CCF. The variance of CCF estimates can be calculated as in Materials and methods, Variance of CCF. In the following we turn to the question of how to determine which scenario applies.

#### Joint distribution of (p, f) and scenario identifiability

By definition, *f* and *p* are both bounded by (0,1). In any tumor, however, the possible range of *f* is constrained by *p* as well as by the sCNA type and the individual scenarios. For example, in scenario **B** of amplification, the mutation occurs in a subset of sCNA-bearing cells, thus *f* is always less than *p* (in this case it is always less than 0.5 *p*). As we show below, the attainable joint distributions of (*p*, *f*) differs among different scenarios and, importantly, this offers the possibility to infer the most likely scenario for a given sCNA-mutation pair based on their observed (*p*, *f*) values. Further, some ‘zones’ of the (*p*, *f*) space overlap with multiple scenarios, thus if the observed (*p*, *f*) fall into these zones, it is impossible to unambiguously identify the exact evolutionary scenarios. Even then, however, because different scenarios sometimes have the same expression of CCF as a function of (*p*, *f*), CCF may still be uniquely estimated. In the following we derive the scenario-dependent (*p*, *f*) joint distributions using heterozygous amplification as example.

In **A**_**1**_, for a given *p*, the observed *f* of the mutation depends on the relative abundance of the r_0_ and r_1_ populations (Figure 3). When r_0_ = 0, the mutation occurred so early that all the diploid cells carry the mutation and belong to the r_1_ subpopulation. *r*_1_ = 1 - *p*, and *f* reaches its upper limit:

8 $$ {f}_h^{A_1}=\frac{1-p+2\times p}{n_t}=\frac{1+p}{2+p} $$

where *n*_*t*_ =2 × (1 - *p*) +3 × *p*, is the averaged total copy number for the sCNA. On the opposite end of the situation is r_1_ = 0, when the sCNA occurred immediately after the mutation such that none of the diploid cells carries the mutation. The lower limit of SAF is reached:

9 $$ {f}_l^{A_1}=\frac{2p}{2+p} $$

**Figure 4:**
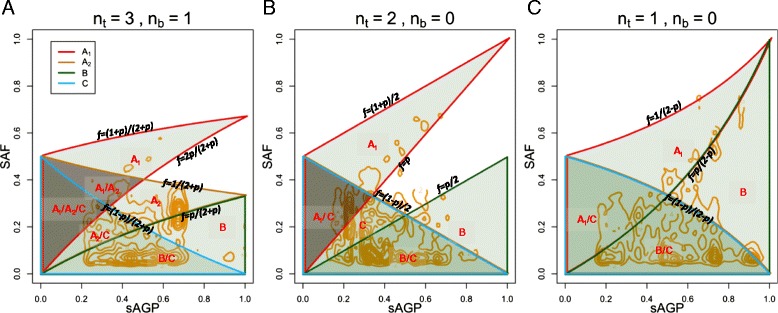
**Identifiability zones in sAGP-CCF space, for amplification (A), CN-LOH (B) and deletion (C), with up to four scenarios described in the main text.** Theoretically permissible areas of sAGP-CCF for different scenarios are marked by borders of different colors, and labeled with a single letter (such as ‘A_1_’) for uniquely occupied zones, and by two or more letters (‘A_1_/C’) for overlapping zones. Regions of light gray support a unique CCF expression, whereas the regions of dark gray cannot unambiguously estimate CCF. The density contours (in orange) depict the distribution of 3,382 mutations in amplification regions (A), 2,008 in CN-LOH, and 4,662 in deletions representing 201 breast tumor samples with least data loss in sAGP estimation. Variants with coverage lower than 20 or SAF smaller than 0.05 were discarded. Only a very small portion of the mutations fall outside the theoretically predicted zones. Among the rest, approximately 48% belong to a unique scenario, but approximately 93% have a unique CCF estimation.

If we plot the possible (*p*, *f*) combinations in an *p-f* plot with *f* on the vertical axis, under scenario **A**_**1**_, the observed f is bounded by (2p/(2 + p), (1 + p)/(2 + p)), where *p* ∈ (0, 1), forming the zone marked **A**_**1**_ in Figure 4A.

For **A**_**2**_, we similarly obtain:

10 $$ {f}_h^{A2}=\frac{1-p+p}{n_t}=\frac{1}{2+p} $$

11 $$ {f}_l^{A2}=\frac{p}{2+p} $$

The observed *f* for A2 is bounded by (p/(2 + p),1/(2 + p)).

For **B**, *f* depends on the relative abundance of the r_1_ and r_2_ populations, and the expressions are

12 $$ {f}_h^B=\frac{p}{n_t}=\frac{p}{2+p} $$

13 $$ {f}_l^B=0 $$

Thus *f* is bounded by [0, p/(2 + p)].

For **C**, the upper limit of *f* is reached when r_0_ = 0, *r*_1_ = 1 − *p*, and

14 $$ {f}_h^C=\frac{1-p}{n_t}=\frac{1-p}{2+p} $$

15 $$ {f}_l^C=0 $$

The *f* is bounded by [0, (1-p)/(2 + p)].

The results for CN-LOH and deletion are described in Materials and methods, CCF estimation and scenario identifiability for CN-LOH and deletion, and shown in Figure 4B and C.

To state the full estimation procedure: when (*f*, *n*_*b*_, *n*_*t*_, *p*) are known for a mutation-sCNA pair, if the (*p*, *f*) combination identifies a unique scenario according to Figure 4, CCF is calculated using Equations (5), (6), and (7). If the (*p*, *f*) combination overlaps with multiple scenarios, CCF may still be calculated if the expressions are the same across the undistinguishable scenarios. Lastly, when the CCF expressions are different among the applicable scenarios, CCF cannot be uniquely determined, however its two or more possible values can still be obtained as valid alternatives. In implementation, as SAF is a random variable with confidence level depending on read depth, there is always uncertainty as to which scenario the observed (*p*, *f*) belongs. We formally calculate the probability of each scenario as described in Materials and methods, Probabilistic scenario identification.

#### Validation, implementation, and performance

**Figure 5:**
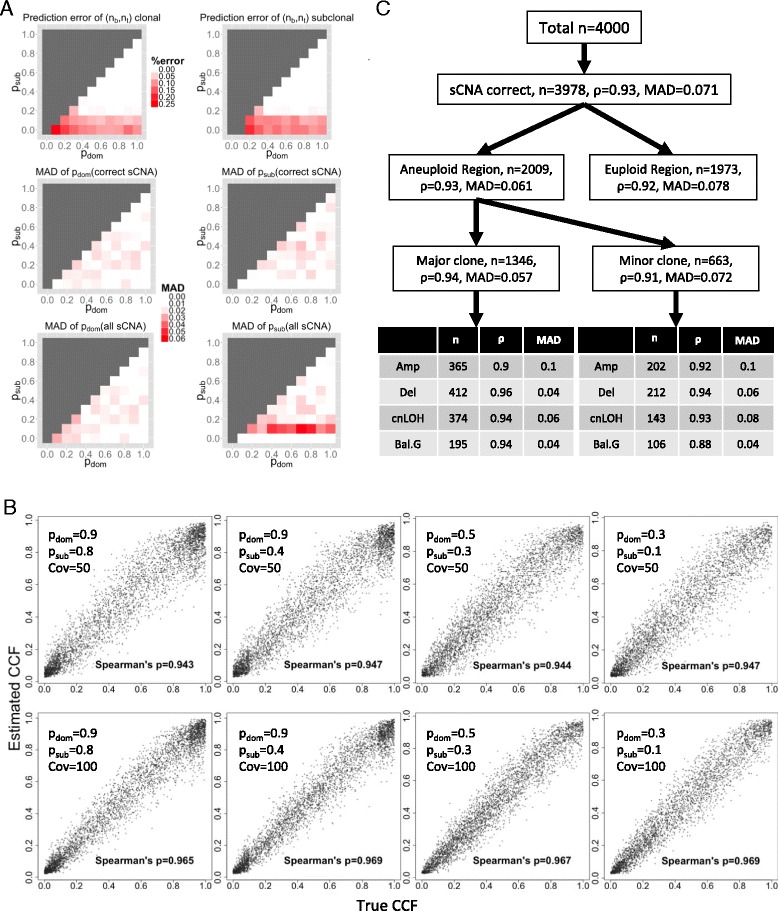
***In silico*** **validation of** ***CHAT*** **performance. (A)** Performance of sAGP inferences. Upper row: percent of error in estimated n_b_ or n_t_, for the dominant (left) and subclonal sCNAs (right), as described in Materials and methods, Performance of sAGP inference. Middle row: the median absolute difference (MAD) between estimated and simulated sAGP values for sCNAs with correctly identified (n_b_, n_t_), or for all sCNAs (Bottom row). The p_sub_ =0 row of the lower-right and middle-right panels had zero error because when p_sub_ =0 there is only one clone in the tumor population and all subclonal sCNA segments have correctly estimated sAGP =0. **(B)** Performance of CCF inference. Shown are scatter plot of simulated and estimated CCF for four p_dom_ - p_sub_ cases and two coverage values: Cov =50 (upper panels) and 100 (lower panels). **(C)** Comparison of CCF inference accuracy among different SNV categories: euploid vs. aneuploidy regions; and in the latter, between the dominant and the minor clones. Lastly, SNVs were divided by sCNA types. The tested case has the following parameter settings: p_dom_ =0.9, p_sub_ =0.6, coverage =50, number of SNV sampled =4,000, number of sCNA sampled =200. ρ, Spearman’s correlation coefficient between the true and the estimated CCF values. MAD: median absolution difference between the true and the estimated CCF values.

To assess the performance of *CHAT* we simulated sCNA and SNV data for a range of copy number configurations, sAGP values, evolutionary scenarios, and CCF values. Details of the simulation procedures were described in Materials and methods, *In silico* validation and computation performance. For sCNAs, we evaluated the performance by reporting: (1) percent of cases of mistaken estimation of sCNA configuration (error in either n_b_ or n_t_) (Figure 5A, top row) for dominant and minor clonal events; and (2) the median absolute deviation of the estimated sAGPs from the known sAGP values for the dominant and minor clones, for either the segments with correct (n_t_, n_b_) identification (Figure 5A, middle row), or all segments (Figure 5A, bottom row). With all of these performance metrics, the errors are the largest when the clonal or subclonal sAGPs are small. The overall errors are small in most situations, suggesting that *CHAT* worked well in recovering the sAGP, n_b_, and n_t_ values. For SNVs, we compared the estimated and the true CCF values in Figure 5B. Across all cases with different coverage and sCNA subclonal parameter settings, the Spearman’s rank correlation coefficient between the known and the estimated CCF values was in the range of 0.946 to 0.97, indicating that *CHAT* makes accuracy CCF inference. To compare performance among SNVs in different sub-categories, we separated those falling in euploid regions from those in sCNAs, and for the latter, separated those in the major and minor subclone events, and those in different copy number status. As shown in Figure 5C, the error rates are similar across these sub-categories, not affected by dominant/minor clonal events or different sCNA types.

*CHAT* is written in R [47] and available as a CRAN package. It can use SNP array-based copy number data and sequencing-derived mutation data, or can use sequencing data as a single input source. It is computationally efficient, taking approximately 1 CPU-hour to analyze every five tumor-normal samples profiled with 850 K SNPs genotying and exome sequencing at 30× (Materials and methods, Computational requirements).

#### Comparison with previous methods

**Figure 6:**
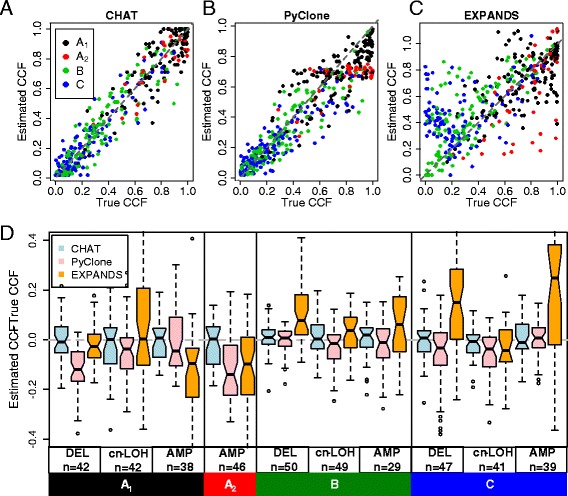
**Performance comparisons of** ***CHAT*** **,** ***EXPANDS*** **, and** ***PyClone*** **. (A, B, C)** Scatter plot of the CCF values estimated by the three methods against the known CCF values used in the simulation. The four lineage scenarios were distinguished by different symbol colors. **(D)** Boxplot showing the estimation errors for each of the three methods, stratified by both sCNA types and lineage scenarios, with the actual number of mutations simulated for each CNA type-scenario combination marked at the bottom of each panel. The sCNA types include heterozygous deletions (A/B), cn-LOH (AA/BB), and amplification (AAB/ABB). Mutations in balanced amplifications (n =55) were not shown in order to reduce clutter.

We compared *CHAT* with two other methods, *EXPANDS* and *PyClone*, that also estimate cellular frequencies for somatic mutations. We simulated 488 mutations that reside in sCNA regions and correspond to different linear scenarios and copy number states (details described in Materials and methods, Comparison with *EXPANDS* and *PyClone*). Figure 6A, B, andC plots the CCF estimated by the three methods against the true CCF used to simulate the observed read counts. *CHAT*-based estimates have the highest correlation with the true CCF (Pearson’s r =0.96), followed by *PyClone* (r =0.94) and *EXPANDS* (r =0.70). As the four lineage scenarios were distinguished by different symbol colors, it can be seen that *PyClone* underestimates CCF in scenarios A_1_ and A_2_ (Figure 6B), likely due to the assumption that mutation and sCNA always co-occur. *PyClone* performs similarly to *CHAT* in scenarios B and C. Like *PyClone*, *EXPANDS* also fails to consider the sCNA-free cells carrying the mutation, thus underestimates CCF in scenarios A_1_ and A_2_. *EXPANDS* overestimates CCF in B and C (Figure 6C) because it ignores B and C by applying A_1_ or A_2_ instead. To assess the impact of CNA status we further stratified the simulated mutations by individual combinations of lineage scenarios and CNA states, including deletion (genotype A/B), copy-neutral LOH (AA/BB) and amplification (AAB/ABB) (Figure 6D). *PyClone* actually has a slight underestimation in scenario B for CN-LOH and amplifications. The overestimation by *EXPANDS* in scenario C only occurs for deletions and amplifications. Overall, *CHAT* has the least bias and least variance in most combinations. Moreover, *CHAT* is the most efficient. It took *CHAT* approximately 1 s to analyze the 488 somatic mutations. *EXPANDS* needed 732 s, and *PyClone* took 4,320 s.

### Application to human breast cancer

We applied *CHAT* to estimate sAGP for sCNAs identified using Affymetrix 6.0 single nucleotide polymorphism (SNP) array data for tumor and germline DNA samples from 732 breast cancer patients [14]. Of these, 445 also have whole-exome sequencing data available, and we estimated CCF for SNVs.

#### sAGP distribution

We detected sCNAs using circular binary segmentation [48] of LRR and BAF data [18], resulting in the identification of an average of 261 sCNAs per tumor (range: 1 to 3,537). The median size of all sCNAs is 1.7 Mb (range: 2.5 Kb to 245 Mb). On average, each tumor carries 125 sCNAs larger than 5 Mb, a size corresponding to approximately 1,500 SNP markers in the 850 K SNP array. Given this sCNA size range, we re-calculated sAGP for genomic bins containing 500 heterozygous SNPs in the germline DNA, a bin size that is approximately 5 Mb, resulting in 502 bins per sample (range: 404 to 794) and constructed the sAGP histogram for every tumor. Eighty-seven tumors (12%) had sCNAs for <50 bins, too few for analyzing the sAGP distribution patterns. For the remaining 645 tumors we fit the sCNAs distribution to either a uni-modal + uniform distribution or a multi-modal distribution using methods described in Materials and methods, Statistical modeling to infer macroscopic clonal structure. In the example in Figure 2C, a three-mode distribution provides the best fit, with sAGP peaks around 0.5, 0.4, and 0.2. The highest peak corresponds to the black-colored sCNAs in Figure 2A and B, while the second and third peaks correspond to the red- and green-colored sCNAs, respectively. In total we observed 392 samples (61%) with best fit by the multi-modal distribution, while 253 (39%) follow the uni-model + uniform distribution. This shows that a majority of the breast tumors analyzed by TCGA contain more than one recognizable aneuploid population, suggesting that the co-existence of more than one subclone is very common.

#### sAGP-CCF joint distribution for known cancer genes

The 445 tumors with both SNP array and sequencing data have an average of 311 somatic mutations per tumor with CCF values (range: from 15 to 4,235, after counting the 8.8% loss due to sCNAs with un-estimable sAGP). While 48% of these mutations fall into a zone with overlapping scenarios, 93% of them have a unique mathematical expression and can produce a valid CCF estimate (Additional file 1: Figure S2). The remaining 7% are assigned missing CCF values due to scenarios with conflicting CCF estimates.

**Figure 7:**
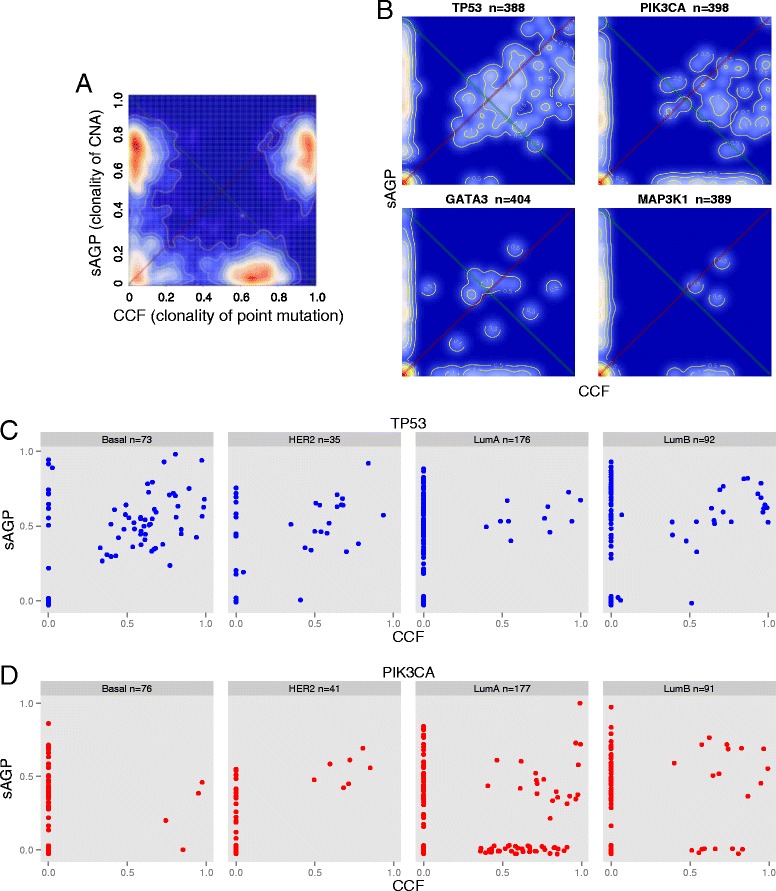
**Comparison of clonality for SNVs and sCNAs of single genes in sAGP-CCF joint distribution heatmaps (A, B) or scatter plots (C, D). (A)** sAGP-CCF 2D heatmap for a hypothetical sample, showing characteristic density peaks as explained in the main text. **(B)** Actual heatmaps for four breast cancer-related genes: *TP53*, *PIK3CA*, *GATA3*, and *MAP3K1*. Sample counts vary by gene, according to how many were removed due to missing values for either sAGP or CCF. **(C, D)** Scatter plot of sAGP-CCF for *TP53* and *PIK3CA*, divided by PAM50 gene expression subtypes. We excluded seven Normal-like samples due to low count.

The calculation of sAGP for most sCNAs and CCF for most SNVs makes it possible to examine the joint distribution of clonality for these two types of genome aberrations. A ‘CCF vs. sAGP’ plot can be created for all copy number and mutation events in a single tumor, or for events affecting a single gene of interest across many tumors. For a given gene, if a sample does not have any somatic mutation in the gene, we assign CCF = 0. Likewise if the copy number of the gene is normal, we assign sAGP = 0. Figure 7A shows a heatmap depicting the density of CCF and sAGP joint distribution for all events in a hypothetical sample (or for a hypothetical gene across all samples). In this two-dimension space, the ‘hot’ peak near the origin (0,0) is typical for most genes, affected by neither somatic mutation nor sCNA. The peak in the upper left (near the sAGP-axis) contains genes with highly clonal CNAs but carrying either no mutation or mutations of low clonality. A plausible interpretation is that for some of these genes, sCNA is a possible driver event. Similarly, the peak at the lower left (near the CCF-axis) contains genes with highly clonal somatic mutations and low-clonality sCNAs. Lastly, genes in the upper-right peak have both high sAGP and high CCF values, suggesting that both copy number changes and somatic mutations may have occurred at very early stages of tumor development, and their joint appearance may be necessary to act as a driver event.

Figure 7B allows close inspection of relative clonality between sCNA and mutations for four genes known to be related to breast cancer [14]: *TP53*, *PIK3CA*, and *GATA3*, which occurred in >10% of analyzed breast tumors, and *MAP3K1*, which had mutations enriched in the luminal A subtype. For *TP53*, there are two noticeable high-density ‘zones’ in the heatmap: one along the sAGP-axis, the other at the upper right, indicating two groups of tumor samples: *TP53* CNA-only and *TP53* CNA/mutation, respectively. This pattern, when stratified by the four PAM50 subtypes [14,49] (Figure 7C), shows that the *TP53* CNA/mutation group is enriched in the Basal and HER2 subtypes (accounting for 72 of 94 Basal and HER2 tumors), whereas the *TP53* CNA-only group is enriched in the Luminal-A (94 of 105), and to a lesser degree, the Luminal-B subtypes (44 of 67). In comparison, the other three genes have not only the CNA-only and CNA/mutation groups, but also a third, mutation-only group near the CCF-axis. Figure 7D shows that for *PIK3CA*, the mutation-only group occurs almost exclusively in the Luminal-A and -B subtypes.

**Figure 8:**
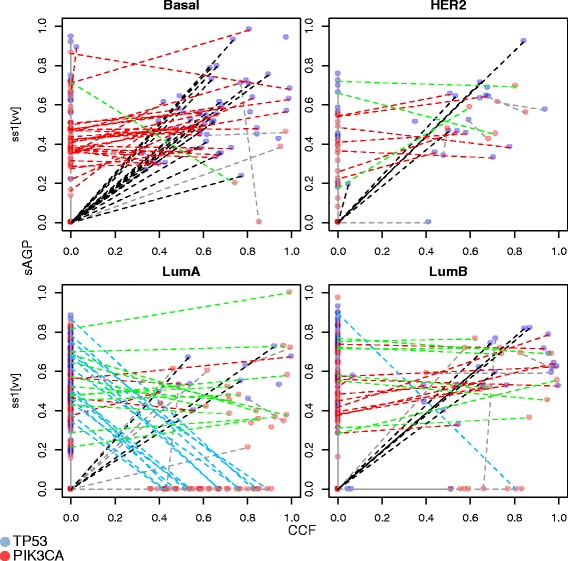
**Two-gene comparison of sAGP-CCF distribution, with values for** ***TP53*** **and** ***PIK3CA*** **in the same samples linked by lines, stratified by PAM50 gene expression subtypes.** Groups with interacting co-occurrence patterns are shown by different line colors. Black: high CCF and sAGP in *TP53*, low in *PIK3CA*. Red and green: high sAGP for both genes, high CCR for *TP53* (red) or high CCF for *PIK3CA* (green). Blue: high sAGP for *TP53* and high CCF for *PIK3CA*.

The CCF-sAGP plot can also be used to compare the clonality distribution between a pair of genes. In Figure 8, *TP53* and *PIK3CA* are shown in red and blue symbols, respectively, with the lines linking the two genes for the same samples. There are three notable patterns of *TP53*-*PIK3CA* clonality. First, samples marked by the black lines have both sCNA and mutation in *TP53* but no aberration in *PIK3CA*. Second, samples marked by the red and green lines tend to have sCNA for both *TP53* and *PIK3CA* and at comparable sAGP, but only mutation in *TP53* (red lines) or *PIK3CA* (green). Third, samples marked by the blue lines had high clonality for *TP53* CNA, but not its mutation, and high clonality for *PIK3CA* mutation, but not its CNA, suggesting co-occurrence of aberrations of these two genes but involving different variant types. These patterns are subtype-specific: Pattern 1 is enriched in the Basal subtype (OR = 4.6 compared to the other three subtypes, *P* = 0.0001 by Fisher’s exact test, for red; OR = 1.2, *P* = 0.67, for green), so is Pattern 2 (OR = 5.3, *P* = 6.4e-8,). Most remarkably, Pattern 3 is almost exclusively found in the Luminal A subtype (OR = 56, *P* = 4.4e-9).

## Discussion

In this work, we developed a computational framework to estimate clonality for both sCNAs and SNVs. It is built on previous methods both by us [18] and by others [32,38,41,43,50]. While *CHAT* does not solve all the issues facing the cancer genome deconvolution problem, it attempts to overcome several important compromises or simplifying assumptions that underlie other methods. First, *oncoSNP* [51] and *THetA* [41] are designed to estimate sCNA clonality, but they do not address the clonality of somatic mutations. Second, Ding et al. [52] used a kernel density estimation method to characterize somatic mutations, but only focused on those in the euploid regions of genome, staying clear of the complicated relationship between SNV and sCNAs. Third, *ABSOLUTE* infers clonality for both sCNA and mutations but only designate subclonal status of the events, stopping short of quantitative estimation. This method was extended in Landau et al. [32] to estimate CCF for somatic mutations even if they are subclonal, but the algorithm only considers the case where sCNA occurred before SNV, equivalent to our scenario B (Figure 3 and Additional file 1: Figure S1), and further assumes that the copy number was altered by only one in the sCNA. In this regard, *CHAT* considers a wider array of possible scenarios. Fourth, *EXPANDS* [50] works with next-generation sequencing data and jointly estimates the absolute DNA copy number, clonality of somatic mutations, and that of sCNAs. However, this method only considers scenario A, and without the r_1_ population. In effect, it assumes that the mutation and sCNA occur at the same instance and are in phase. These assumptions explained its underestimation of CCF in scenario A and overestimation in B and C (Figure 6). Fifth, *PyClone* [43] infers clonality of somatic mutations and performs phylogenetic analysis. It receives as input the integer copy number profiles estimated from other methods, but only considers scenarios A and B, disregarding the possibility of a branching lineage (scenario C). Furthermore, for scenario A, it assumes co-occurrence of SNV and sCNA, thus ignoring the r_1_ population. In sum, the first key contribution of *CHAT* is in providing a general mathematical framework that enumerates the complete set of scenarios covering the possible order and phase of the sCNA and the mutation.

Like many of the previous methods, *CHAT* has its own limitations, primarily in being unable to resolve extremely complex events such as three-way mixing or above. It models two-population mixing at each genomic region (a gene, a sCNA, or a bin) and works best when the tumor has not experienced extensive and repeated copy number alterations. In the TCGA breast tumor dataset we found that 9.3% of sCNAs do not follow the regional two-way mixing model and preclude sAGP estimation. For the other, permissible sCNAs, *CHAT* can proceed, and is able to infer the co-existence of two or more subpopulations by analyzing the distribution of sAGP or CCF values. We wish to re-emphasize that while *CHAT* invokes two-way mixing for each individual genomic region, it is not limited to infer the presence of only two populations of cells. Globally, the number of peaks in the sAGP or CCF distribution has no restriction, and can be very high when the signal-to-noise ratio is improved, such as with ultra-deep sequencing data, for example, [39].

A second contribution of *CHAT* is in systematically assessing the input data combinations that leads to ‘unidentifiable zones’, in which the CCF, or ‘scenarios’ (that is, the evolutionary order and phase of the sCNA and SNV), cannot be resolved even with perfect data. Importantly, we found that in many situations, even if the evolution scenario is undetermined, CCF can still be estimated. The ability to objectively evaluate the power of inference in any given dataset is an important part of method development. Our treatment of this topic therefore sets useful constraints for future development of similar analysis tools. We found that, with the TCGA breast tumor data, about 9.3% of the identified sCNAs cannot be explained by local two-way mixing and were assigned missing sAGP values. In the second step, about 7% of the SNVs have unidentifiable CCF because they fall in either an inadmissible sAGP-SAF zone or a region with conflicting CCF estimates. Thus 93% of SNVs are suitable for CCF estimation, despite the fact that 48% of them involve ambiguous scenarios.

When applying *CHAT* to the breast tumor dataset, we found that approximately 61% of the breast tumors in the TCGA cohort contain more than one recognizable sAGP peaks, suggesting that even in a tumor cohort collected for studying inter-tumor diversity there is opportunity to detect intra-tumor mixing of multiple populations of aneuploid cells. And the results show that extensive intra-tumor heterogeneity does exist. This observation expands the earlier view that tumor-normal mixing contributes to intra-tumor heterogeneity, and confirms the results from single-cell and multi-region analyses in other solid tumors, for example, [25,33]. We wish to point out that the estimate of 61% was based on a specific analysis approach, and would vary with alternative parameter choices. For example, by using regularly spaced bins we found that 392 of 645 samples (61%) were multi-modal, yet by using the naturally occurring sCNAs, only 635 samples had sufficient number of events, and 373 of them, or 59%, were multi-modal. More notably, of the 392 multi-modal samples called with bins, and the 373 multi-modal samples called with sCNAs, the overlap is 235, or about 60% for either method. This level of concordance is related to the inherent shortage of observations for many samples: when the number of sCNAs or bins is in the 50 to 100 range, and if the primary peak is far larger than the secondary peak, the inference is less stable. These data-derived limitations can be overcome in the future when more samples are analyzed with whole-genome sequencing, which will likely yield a far greater number of genealogically informative markers.

A useful downstream analysis of the inferred clonality measures is to assess the distribution patterns of cellular frequencies of somatic aberrations, and to detect frequency clusters when they do exist for a given tumor. *CHAT* provides the option of characterizing the macroscopic clonal structure by a cluster-based approach (Figure 1). It is important to emphasize that these frequency clusters, despite their many valid interpretations, cannot be equated to individual subclones. A subclone may carry somatic events in multiple clusters, and may share some events with another subclone if they are descendants of the same parental clone. The full deconvolution of the observed aggregate pattern into those contributed by individual subclones requires further mathematical modeling and involves additional challenges. Several methods have recently appeared to address this ‘Blind Source Separation Problem’, or synonymously, ‘Feature Allocation Problem’ [53-56]. *CHAT* can be applied in tandem with these methods, that is, the sAGP and CCF output from *CHAT* can serve as the input data for further feature allocation to component subclones.

The co-existence of multiple clonal populations in bulk tissues could be explained by several population genomics models that are not mutually exclusive. First, in a multi-region parallel-evolution model, the tumor tissue might contain geographically segregated ‘pure’ populations, reflecting branched evolution of multiple clones of homogeneous tumor cells, each developing a different genomic profile that reflects its cell type of origin and adaptation to the local tissue habitat. This model can only be tested by spatially restricted sampling. Second, even in the absence of spatial segregation, the non-spatial, sequential expansion model could still lead to multiple nested populations. In some episodes, a burst of mutations or copy number variants might occur in one cell, which subsequently expands to a detectable clonal size driven by its unusually high selective advantage [57-59]. Alternatively, even in the absence of such disruptive genomic crisis, the slow, successive replacement of mildly advantageous clones could also result in a series of partial sweeps, leading to co-existence of multiple clones at any given time [60,61]. In other words, episodic acceleration of cancer genome evolution can take place either via mutation rate ‘spikes’ or simply through variabilities of selective advantage among driven events within a constant mutation regime. There are many routes that could lead from gradual evolution to punctuated equilibrium [62] in the history of each cell population; and this temporal heterogeneity is often further compounded in solid tumors by their spatial heterogeneity.

## Conclusion

We developed an automated pipeline that estimates cellular fractions for both sCNAs and mutations, and uses their distributions to inform macroscopic clonal architecture. It considers a wider range of evolutionary scenarios than existing methods concerning the timing and phase relationship between a sCNA and a mutation it contains. Our method also explicitly evaluates model- and parameter- identifiability. When applied to a previously analyzed set of >700 breast tumors we found more than half of the tumors appear to contain multiple recognizable aneuploid tumor clones, and many show subtype-specific differences in clonality between sCNA and mutation in known cancer genes. This method adds to the available toolkit for examining intra-tumor heterogeneity using bulk tumor genomic data.

## Materials and methods

### Data access and sCNA identification

From the Cancer Genome Atlas Data Portal [63] we downloaded: (1) the Level-2 copy number data derived from the Affymetrix Genome-Wide Human SNP Array 6.0 (the ‘XX-byallele.copynumber.data.txt’ files) for 732 breast tumor DNA and their paired normal tissue DNA; and (2) the VCF files for whole-exome sequencing data for a subset of 522 tumor-normal samples analyzed by TCGA [14]. The IDs of the 732 samples are in Additional file 2. Of these, 445 samples have both SNP array and DNA sequencing available. The SNP array data were downloaded on 12 December 2012, while the sequencing data were downloaded on 22 March 2013. Each VCF file contains variant information for both the tumor and the paired normal sample. The procedures for variant calling and identification of somatic variants can be found in the Online Supplementary Methods of [14]. Counts for somatic and reference alleles of both tumor and normal samples were extracted for use in this study.

In addition, we also downloaded the clinical annotation file, including the PAM50 designations of all the involved patients, on 17 December 2012.

sAGP estimation (see below) can be performed on two types of user-selected spatial units: (1) genomic bins, predefined for each sample, typically consisting of 500 heterozygous markers in the germline DNA; (2) naturally observed sCNA segments, which we detect using the Circular Binary Segmentation (CBS) method [48], as follows. We independently perform segmentation on the LRR and the folded BAF (absolute value of BAF minus 0.5) values, using default parameters in the R package *DNAcopy* [46], except that ‘minimal markers required’ was set to 5. With CBS results for both LRR and BAF, the two sets of change points are merged as follows: if a BAF change point falls within 5 markers of an LRR change point, either upstream or downstream, it is removed, that is, only the LRR breakpoint is kept, under the assumption that the two change points capture the same event, but the BAF change point is less accurately placed due to the greater sparsity of heterozygous markers.

After merging, the mean of LRR and folded BAF values are computed for each DNA segment (or the bin) in each sample, and used as input data for AGP and sAGP inference in the next step. For binned files, the bin length is on average 5.1 Mb, and each sample has an average of 502 bins.

### sAGP inference

As discussed in the main text, we jointly use BAF and LRR values to estimate sAGP for each sCNA, under the assumption of regional two-way mixing. The algorithm has three steps:

i. Data pre-processing

We assume the allele-specific copy number data are already in bi-allelic format, with the following fields in the input file: SNP ID, chromosome, position, A allele count, B allele count. To note, the allele counts may not be integer numbers, but could be real-numbered values from the original CEL file. SNP markers are first grouped into either bins or merged sCNAs as described above. For each bin/sCNA, the median LRR and median folded BAF are calculated, and a segmentation file containing the above information for each segment is generated for each sample.

In the initial normalization of SNP array data the absolute LRR values depend on the genome-wide average ploidy, which is affected by the relative abundance of different copy number states in the genome. For example, in a tumor with a high fraction of cells undergone genome-wide doubling, the DNA segment located near the origin of the BAF-LRR plot are AABB, instead of the normal diploid configuration AB, and the global ploidy can be well above 2. The first step of sAGP estimation is therefore to ascertain the genotype of the sCNAs near the origin, following the procedures described in [18]. This allows unambiguous assignment (when possible) of copy number states for other sCNAs in the genome and the calculation of average ploidy. The deviation of BAF and LRR values of the baseline sCNAs from (x_0_,y_0_) is also used to quantify $s{d}_{BAF}^2\kern0.5em  and\kern0.5em s{d}_{LRR}^2$ for use in downstream analysis.

ii. Estimate sAGP and absolute copy numbers

The method we used to estimate sAGP is extended from our AGP inference algorithm. For a sCNA with copy number configuration (n_b_,n_t_), where n_b_ is number of minor allele, and n_t_ number of total alleles, when mixed with a balanced diploid population its theoretical BAF and LRR values are:

$$ BAF=\left|\frac{p\times {n}_b+1-p}{p\times {n}_t+2\times \left(1-p\right)}-0.5\right|+{x}_0 $$

$$ LRR=lo{g}_2\left(p\times {n}_t+2\times \left(1-p\right)\right)-1+{y}_0 $$

where *p* is sAGP, and x_0_, y_0_ are the coordinates of the (n_t_ =2, n_b_ =1) state. When *p* changes, the points (BAF, LRR) follow a family of curved lines on the BAF-LRR plot, starting from the origin (x_0_,y_0_). Each line corresponds to a unique combination of (n_b_, n_t_) and is called a canonical line; and each point on this line uniquely corresponds to an sAGP value. The main task is to assign each observed segment to a canonical line. Due to noise, a sCNA does not locate precisely on a canonical line. Thus for each sCNA, we scan all possible canonical lines to find the one satisfying the following criteria:

- Distance to the closest canonical line ≤2 $\sqrt{s{d}_{BAF}^2+s{d}_{LRR}^2}$; where *sd*^*2*^_*BAF*_, and *sd*^*2*^_*LRR*_ are the estimated standard errors of BAF and LRR values.

Sometimes multiple lines satisfy (a) and result in multiple sAGP and n_t_ estimates. In such cases we apply

b. Choose *sAGP* = *argmin*(*F* = *n*_*t*_ − *ploidy* + |*p*_*s*_ − *p*|); where p_s_ is sample-wide AGP and *ploidy* is the estimated global average ploidy from step ii). This criterion chooses the most probable canonical line as the one that results in a total copy number close to the genome-wide ploidy and an sAGP close to the global AGP.

If no canonical line can be found in (a), that is, the deviation is greater than the specified 2× scale of the standard deviations of BAF and LRR markers, we consider the sCNA not meeting the regional two-way mixing hypothesis, and its sAGP is assigned NA, its n_b_ and n_t_ are also treated as missing values in downstream analysis.

### Statistical modeling to infer macroscopic clonal structure

As explained in the main text, sAGP values can be calculated for either predefined genomic bins or identified sCNAs. In the per-bin analysis, the user can choose to filter out the non-sCNA bins or those with very small sAGP values, as true sCNAs with length shorter than the bin width tend to have reduced sAGP estimates due to the flanking euploid regions. In our analysis of the breast tumor data we applied two filtering steps. First, we considered bins with median folded BAF ≤0.04 and absolute median LRR ≤0.16 to be euploid, and assigned sAGP =0. Second, before sAGP clustering, we removed bins with sAGP ≤0.05 to remove the contribution of the small sAGP values. At this step there is an average of n =224 bins left per sample. The two models described in the main text are evaluated in a maximal likelihood framework. For Model-1, the log likelihood has a uniform and a normal component:

$$ l={\displaystyle \sum_{i=1}^n} \ln \left(\frac{A}{range\left(\boldsymbol{Y}\right)}+\left(1-A\right)\times Norm\left({y}_i,\mu, \sigma \right)\right) $$

where **Y** is the observed sAGP vector for a given sample, with components y_i_, i =1,2,…,n, where n is the number of DNA segments after filtering. A is a scalar so that A/range provides the scaled uniform distribution. μ and σ are the mean and standard deviation of the single peak in the model following the normal distribution. We constrain A and μ in the range (0, 1). The parameters A, μ, and σ are estimated using the maximum likelihood approach, implemented in customized scripts (part of *CHAT*) written in the R statistical programming language [45].

Model-2 is fitted using a Dirichlet process Gaussian mixture model to infer the uncertain number of peaks and their relative abundances. The parameterization is as follows:

$$ {y}_i\Big|{\mu}_i,{\sigma}_i\sim Norm\left({\mu}_i,{\sigma}_i\right),\ i=1,2\dots, n $$

$$ {\mu}_i,{\sigma}_i\Big|G\sim G $$

$$ G\Big|\alpha, {G}_0 \sim DP\left(\alpha {G}_0\right) $$

$$ {G}_0= Norm\left(\mu \Big|{\mu}_1,\sigma /{k}_0\right) InvWishart\left(\sigma \Big|{\nu}_1,{\psi}_1\right) $$

$$ {G}_0= Norm\left(\mu \Big|{\mu}_1,\sigma /{k}_0\right) InvWishart\left(\sigma \Big|{\nu}_1,{\psi}_1\right) $$

$$ {k}_0\Big|{\tau}_1,{\tau}_2\sim \Gamma \left({\tau}_1/2,{\tau}_2/2\right) $$

Together these expressions describe a standard Dirichlet process mixture of normal model [40]. The implementation of the MCMC fitting is via R package *DPpackage* [56]. There are different ways to specify the prior parameters for the normal mixture model. The baseline Gaussian distribution G_0_ relies on three prior parameters, μ_1_, σ, and *k*_0_, where σ is explicitly modeled by an Inversed Wishart distribution with priors ν_1_ and *ψ*_1_, and *k*_0_ follows a Gamma distribution. In practice, the hyperpriors, ν_1_, *ψ*_1_, and k_0_ can also be allowed to be random variables with a given prior distribution, and the model will have higher power to fit minor peaks in the data. In this work we used a conservative setting of prior parameters in terms of peak discovery sensitivity.

Model-1 cannot be included as a special case of Model-2, since when y is truly uniformly distributed, Dirichlet process tends to call multiple peaks instead of one peak, even with current conservative prior setting. Our solution is to fit both models, then numerically compute the likelihood of each model, and use Bayesian Information Criterion (BIC) to select the better model. Model-1 has three free parameters: A, μ, and σ, while Model-2 has seven: *a*_0_, *b*_0_, *k*_0_, ν_1_, *ψ*_1_, τ_1_, and τ_2_.

### CCF estimation and scenario identifiability for CN-LOH and deletion

In the main text we presented how *CHAT* performs CCF estimation for the case of hemizygous amplifications (n_b_ =1, n_t_ =3). While this sCNA type has all four scenarios, Scenario **B** is not available for some other types of sCNAs, including LOH (n_b_ =0, n_t_ ≥1) and balanced allelic gains (n_b_ >1, n_t_ =2n_b_). Below we will describe the cases of copy neutral LOH (CN-LOH) and hemizygous deletion.

#### CN-LOH

In Scenario **A**_**1**_ (Additional file 1: Figure S1A)

$$ {r}_0+{r}_1+{r}_2=1 $$

$$ {r}_2=p $$

$$ f=\frac{r_1+{n}_a\times {r}_2}{n_t}=\frac{r_1+2\times {r}_2}{2} $$

where *n*_*t*_ =2 × (1 - *p*) + *n*_*t*_ × *p* =2 and *CCF* = *r*_1_ + *r*_2_. Using the above equations it is easy to show that

$$ CC{F}^{A_1}\left(f,{n}_b,{n}_t,p\right)={n}_t\times f-p\times {n}_a+p=2\times f-p $$

Scenarios **A**_**2**_, **B**, and **C** have the same expression:

$$ CC{F}^{A_2}=CC{F}^B=CC{F}^C=2f $$

Note that **A**_**2**_ and **C** not only have the same expression for CCF, they also have the same three-population composition, although the three populations emerge by different evolutionary routes. A previous study [32] failed to take **A**_**1**_ into consideration and could have overestimated CCF under **A**_**1**_, thus could have designated subclonal mutations as clonal when **A**_**1**_ is the true evolutionary scenario.

The lower and upper limits of SAF in each scenario can be derived using the same process as in the main text. In scenario **A**_**1**_, *f* reaches its upper limit when r_0_ = 0, and r_1_ = 1-r_2_.

$$ {f}_h^{A_1}=\frac{1-p+2\times p}{n_t}=\frac{1+p}{2} $$

On the opposite side is r_1_ is zero, when *f* reaches its minimum value:

$$ {f}_l^{A_1}=\frac{2p}{2}=p $$

With *p* takes values from (0,1), the areas defined by these limits are shown in Figure 4B.

For scenario **B**:

$$ {f}_h^B=\frac{p}{N_t}=\frac{p}{2} $$

$$ {f}_l^B=0 $$

And scenario **C**:

$$ {f}_h^C=\frac{1-p}{N_t}=\frac{1-p}{2} $$

$$ {f}_l^C=0 $$

#### Hemizygous deletion

All four scenarios have the same expression:

$$ CC{F}^{A_1}=CC{F}^{A_2}=CC{F}^B=CC{F}^C=f\left(2-p\right) $$

Similar to CN-LOH, **A**_**2**_ and **C** not only have the same expression for CCF, they also have the same three-population composition.

The upper and lower limits for this sCNA type are:

$$ {f}_h^{A_1}=\frac{1-p+p}{N_t}=\frac{1}{2-p} $$

$$ {f}_l^{A_1}=\frac{p}{2-p} $$

$$ {f}_h^B=\frac{p}{N_t}=\frac{p}{2-p} $$

$$ {f}_l^B=0 $$

$$ {f}_h^C=\frac{1-p}{N_t}=\frac{1-p}{2-p} $$

$$ {f}_l^C=0 $$

The areas defined by these limits are shown in Figure 4C.

### Variance of CCF

We use the same approach as described in [32] to estimate the standard deviation of CCF. The distribution of CCF is modeled as Binomial:

$$ \Pr \left(CCF=x\right)\propto Binomi\left(S\Big|N,G\left(x,p,\Theta \right)\right) $$

where S is the read count for the somatic allele and N is the total read depth. *G*(•) is expected value of SAF given CCF value *x*, sAGP value *p*, and lineage scenario Θ. *G* is simply obtained by reversing the CCF expressions described in the main text (Equations (1), (2), (3), (4), (5), (6), and (7)). We assume a uniform prior on *x* and the expectation and variance of CCF can be calculated as:

$$ EXP(CCF)=\frac{{\displaystyle {\int}_0^1} Binom\left(S\Big|N,G\right)xdx}{{\displaystyle {\int}_0^1} Binom\left(S\Big|N,G\right)dx} $$

$$ Var(CCF)=\frac{{\displaystyle {\int}_0^1} Binom\left(S\Big|N,G\right){x}^2dx}{{\displaystyle {\int}_0^1} Binom\left(S\Big|N,G\right)dx}-EXP{(CCF)}^2 $$

To note, the expectations of CCF are identical to the expressions in the main text (Equations (1), (2), (3), (4), (5), (6), and (7)).

### Probabilistic scenario identification

The task is to use the observed somatic allele frequency (*f*) and sAGP value to determine the most likely scenario among the four scenarios described in the main text. We assume that f has a uniform prior, U(0,1), and we are interested in calculating the likelihood that the sSNV occurred before the sCNA, given the copy number configuration (n_b_, n_t_), known sAGP (p), and the observed allele counts. Let f_0_ denote the true f. The probability of observing S count of the somatic allele is model by Binomial(f_0_, N) and the likelihood of each scenario is the probability of observing S given the scenario is true, integrated over all the possible values of f_0_:

$$ \begin{array}{l}{p}_X=L\left( Scenario\ X\Big|p,{n}_b,{n}_t,N,S\right)= \Pr \left\{S\Big|X,\ p,{n}_b,{n}_t,N\right\}={\displaystyle \int } \Pr \left\{S\Big|{f}_0,N\right\}\times \Pr \left\{f={f}_0\Big|X,\ p,{n}_b,{n}_t\right\}\mathrm{d}{f}_0\hfill \\ {}={\displaystyle \underset{f_l^X}{\overset{f_h^X}{\int }}} \Pr \left\{S\Big|{f}_0,N\right\}d{f}_0\hfill \end{array} $$

where X is **A**_**1**_, **A**_**2**_c scenarios, and f^X^_h_, f^X^_l_ are computed according to Equations (8), (9), (10), (11), (12), (13), (14), and (15) in the main text and those in the section (CCF estimation and scenario identifiability for CN-LOH and deletion) above. We then compute the summation of p_X_:

$$ P={p}_{A1}+{p}_{A2}+{p}_B+{p}_C $$

and normalize each likelihood using *P*:

$$ {\tilde{p}}_X=\frac{p_X}{P} $$

We calculate the normalized probability for each scenario, as well as all the possible combinations of multiple scenarios. For example, the probability of either scenario A1 or C is ${\tilde{p}}_{A1C}={\tilde{p}}_{A1}+{\tilde{p}}_C$. There are in total 2^4^ - 1 = 15 possible combinations. If the normalized probability of any of the four scenarios is greater than 0.95, the SNV is assigned to the corresponding scenario. If none of the single-scenario probability exceeds 0.95, we ask if any of the six two-scenario combinations have probability >0.95. If this step fails, we next examine the four possible three-scenario combinations, and so forth. If all the above steps fail, we report the SNV scenario **A**_**1**_**/A**_**2**_**/B/C**, and no unique CCF can be estimated in this case.

### *In silico* validation and computation performance

#### Performance of sAGP inference

We first tested the performance of *CHAT* in sAGP estimation. We simulated LRR and BAF values for a series of sCNA datasets with two aneuploid tumor populations, which are mixed with the euploid population. The first population is the dominant clone, with an assigned sAGP value of p_dom_ ~ [0.1,0.2,…,1.0]. The second population is a minor clone, with an assigned sAGP value of p_sub_ ~ [0,0.1,…p_dom_-0.1]. The fraction of the euploid population is 1- p_dom_ - p_sub_. In all, there are 55 p_dom_ - p_sub_ combinations; and for each, we simulated 200 euploid segments (n_b_ =1, n_t_ =2, sAGP =0) and 200 sCNA segments, of which 133 (about 2/3) were assigned to the dominant clone (sAGP = p_dom_), and the remaining 67 were assigned to the minor clone (sAGP = p_sub_). Within each clone, the sCNAs were assigned to one of four copy number configurations with the following ratios: 2/7 for deletion (n_b_ =0, n_t_ =1), 2/7 for CN-LOH (n_b_ =0, n_t_ =2), 2/7 for amplification (n_b_ =1, n_t_ =3), and 1/7 for balanced doubling (n_b_ =2, n_t_ =4). The BAF and LRR values were generated using the assigned sAGP and copy number configuration with the following formula:

16 $$ BAF=\left|0.5-\frac{p\times {n}_b+1-p}{n_t}\right|+ Normal\left(0,{\sigma}_{BAF}\right) $$

17 $$ LRR=lo{g}_2{n}_t-1+ Normal\left(0,{\sigma}_{LRR}\right) $$

where p stands for sAGP, and n_t_ is the averaged total copy number for the local segment: 2 (1-p) + n_t_ × p. σ_BAF_ and σ_LRR_ are the standard deviation values of the per-segment BAF and LRR, respectively. For the Affymetrix 6.0 platform, the per-SNP standard deviation for BAF is about 0.05, and for LRR is about 0.25 (our observation). Thus the choice of σ_BAF_*=*0.01 and σ_LRR_ =0.04 is equivalent to the standard error of an sCNA of approximately 36 SNP markers. For a 1 million SNP platform, 36 SNPs cover approximately 110 kb, therefore ours are conservative choices for sCNAs 110 kb or longer, profiled by 1 million SNPs or more.

After generating the BAF and LRR values using Equations (16) and (17) for the 400 segments for each of the 55 p_dom_ - p_sub_ combinations, we applied *CHAT* to estimate sAGP, n_b_, and n_t_ for each simulated segment and reported the results in Figure 5A.

#### Performance of CCF estimation

Of the 55 p_dom_ - p_sum_ combinations described above we selectively tested CCF inference in four cases: p_dom_ - p_sum_ ~ (0.9,0.8), (0.9,0.4), (0.5,0.3), and (0.3,0.1). For each case, we simulated 4,000 SNVs, of which approximately 2,000 fall in the 200 euploid segments, and the other approximately 2,000 fall in the 200 sCNA regions, with the (sAGP, n_b_,n_t_) assignment implemented as described above. In effect we assume that the euploid intervals account for 50% of the genome. To make the test realistic, we used the sAGP, n_b_, and n_t_ estimated by *CHAT* rather than the true values used in the initial simulation of the LRR and BAF data. If the SNV falls in a euploid region, the assigned SAF was randomly drawn from uniform(0, 0.5) and the corresponding ‘true’ CCF = SAF × 2. If it falls in an aneuploid region, we randomly choose the lineage scenario from (**A**_**1**_, **A**_**2**_, **B**, **C**) according to the local copy number configuration. If the sCNA is a CN-LOH or balanced doubling region, we limit the scenarios to (**A**_**1**_, **B**, **C**). The upper and lower limits of the chosen scenario were determined using Equations (8) to (15) in the main text and the equations in Materials and methods, CCF estimation and scenario identifiability for CN-LOH and deletion. SAF values were then randomly drawn from within this permissible range: uniform(f_l_, f_h_), where f_l_ and f_h_ were the lower and upper limits. ‘True’ CCF values were computed using Equations (1) to (7) in the main text. Lastly, from the ‘true’ CCF we simulated the allele counts in two steps. For a mean read depth k, the actual coverage at a given SNV, N, was sampled from N ~ Poisson(*k*). With N and f (that is, CCF) thus assigned, the count of the somatic mutation allele was sampled from Binomial(*f*, N). Based on the estimated sAGP, copy number configuration and the simulated somatic allele counts we used *CHAT* to estimate CCF. The estimated values were compared with the ‘true’ CCF for both k =50 and k =100. For all eight cases (four p_dom_ - p_sub_ combinations and two k values) we calculated the Spearman’s rank correlation coefficient and/or the median absolute deviation (MAD) between the known and estimated CCF values (Figure 5B and C).

#### Computational requirements

We estimated the time and memory requirement of *CHAT* using the TCGA dataset for breast tumors. The time estimate below is based on allele-specific copy number data with 850 K SNPs for tumor-normal pairs and whole-exome sequencing data with approximately 30× average coverage. For binned segmentation (approximately 500 heterozygous SNPs per bin), it takes 2 min to complete the sAGP and CCF estimation for one tumor/normal pair, and it requires about 10 MB memory. For detected sCNAs, the computational time increases to an average of 12 min per sample pair. The above estimation is based on running R scripts with a single processor (AMD Opteron 6136, 2.4GHz with 4G RAM) and counting input file reading time. In *CHAT*, the user can apply the R package parallel to enable multi-thread processing. This allows the use of as many processors as available. On our server (32 AMD Opteron 6136 CPUs and 128G RAM), our test run used 14 processors on average, and it took 10 h (140 CPU-hours) to complete the CBS segmentation, sAGP estimation for 732 breast tumor-normal samples and CCF estimation for 445 samples with downloaded VCF files.

#### Comparison with EXPANDS and PyClone

We simulated 200 CNA regions, with sAGP values randomly drawn from U(0,1), and copy number configurations assigned by the ratio of 2/7 for deletion, 2/7 for CN-LOH, 2/7 for amplification, and 1/7 for balanced doubling, as described in Performance of sAGP inference. For each CNA, the LRR and BAF values were simulated as in Performance of sAGP inference. We then simulated 1,000 somatic mutations evenly across the ‘genome’, with 488 that happened to fall in an sCNA region (with the rest falling in euploid regions). For these 488 somatic mutations, we assigned them to lineage scenarios with similar ratios across A_1_, A_2_-when possible, B, and C. The actual number assigned to each combination was shown in Figure 6D. For each mutation thus assigned, we sampled the somatic allele frequency (f) uniformly from its permissible range as described in Performance of CCF estimation, calculate the corresponding true CCF value, and simulated the sequencing read counts at the average coverage of k =50, as described in Performance of CCF estimation. We applied *CHAT* to estimate (sAGP, n_t_, n_b_) from the simulated LRR and BAF data, then estimated CCF from the simulated read counts, sAGP and the estimated lineage scenarios and (n_t_, n_b_) status. In parallel, we applied *PyClone* to the same dataset, using the simulated read counts and the true (n_t_, n_b_) as input to estimate CCF. The choice of true (n_t_, n_b_) rather than the *CHAT*-estimated (n_t_, n_b_) should slightly favor *PyClone* as the errors in estimating (n_t_, n_b_) are not incorporated. Lastly, we applied *EXPANDS* to estimate CCF, using the simulated LRR and the observed somatic allele frequency as input. We compared the estimated CCF of the three tools with the known CCF in Figure 6. To make Figure 6D less cluttered we omitted balanced amplifications, thus only showing 423 mutations for the other CNA types.

### Data availability

CHAT source package is available at https://sourceforge.net/projects/clonalhetanalysistool/files/?. It is released under fully open source license, GPL (≥2.0). It is also available as a CRAN-R package. The breast tumor data were downloaded from the Cancer Genome Atlas Data Portal as described in Data access and sCNA identification. The TCGA IDs for the 732 tumors are in Additional file 2. The simulated data are available at http://sourceforge.net/projects/clonalhetanalysistool/files/simulations/ and as Additional file 3.

## Additional files

Additional file 1:
**Supplementary figures (Figure S1-S3) and legends describing additional information.**
Additional file 2:
**TCGA Sample IDs for the 732 breast tumors we analyzed in this study.**
Additional file 3:
**Simulated data used to compare** ***CHAT*** **,** ***PyClone*** **, and** ***EXPANDS.***

## Abbreviations

CCF: Cancer cell fraction
*CHAT*: Clonal heterogeneity analysis tool
ICGC: International Cancer Genome Consortium
SAF: Somatic allele frequency
sAGP: Segmental aneuploidy genome proportion
sCNA: Somatic copy number alteration
TCGA: The Cancer Genome Atlas
