# Supplementary material for: A general framework for analyzing tumor subclonality using SNP array and DNA sequencing data
Source: Genome Biol. 2014 Sep 25;15(9):473. doi: 10.1186/s13059-014-0473-4 (PMC4203890; doi:10.1186/s13059-014-0473-4)

Supplementary File 1

Figure S1: Lineage scenarios for CN-LOH (A) and heterozygous deletion (B).

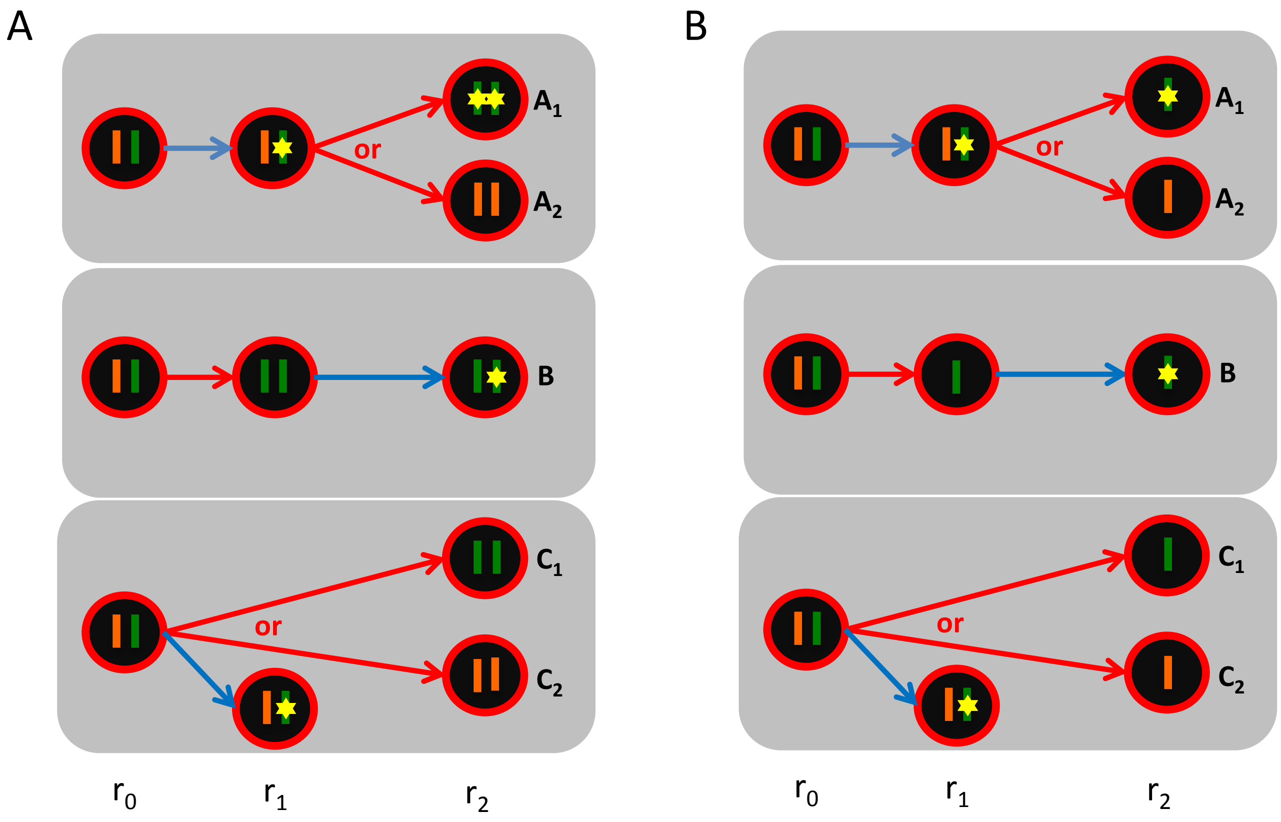

Figure S2: Distribution of the percentage of somatic mutations associated with a unique scenario (black) and the additional percentage with unique CCF estimates (red). From left to right are the results for 445 breast tumor samples, ordered by the unique-scenario percentage.

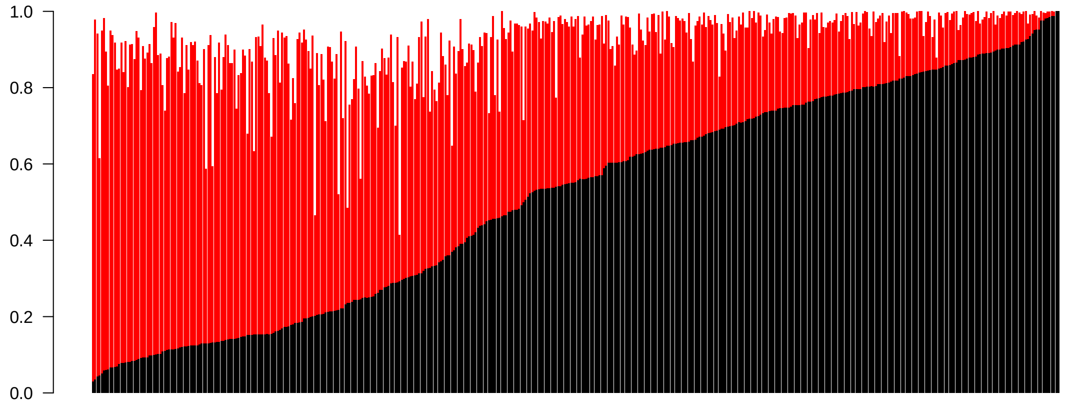

Supplement: Additional file 1: — Supplementary figures (Figure S1-S3) and legends describing additional information. [file 13059_2014_473_MOESM1_ESM.pdf]
